# Chinese herbal medicine for previous cesarean scar defect: A protocol for systematic review and meta-analysis

**DOI:** 10.1097/MD.0000000000023630

**Published:** 2020-12-11

**Authors:** Jiashuai Deng, Sixuan Li, Yangzhi Peng, Zhaoxing Chen, Changhong Wang, Zhipeng Fan, Mao Zhao, Yuchang Jiang, Zhaodi Wang, Yong Jiang

**Affiliations:** School of Basic Medical Sciences, Chengdu University of Traditional Chinese Medicine, Chengdu, China.

**Keywords:** Chinese herbal medicine, meta-analysis, previous cesarean scar defect, protocol

## Abstract

**Background:**

: Previous cesarean scar defect (PCSD) is a gynecological disease that can cause bleeding after intercourse, prolonging menstrual period, intermenstrual bleeding, dysmenorrhea, and even lead to infertility. Chinese herbal medicine plays an important role in the treatment of gynecological diseases in China and East Asia. This study aims to assess the efficacy and safety of Chinese herbal medicine for PCSD.

**Methods:**

: We search the following databases: PubMed, the Cochrane Library, Chinese Biomedical Literature Database (CB), Chinese Science and Technique Journals Database (VIP), EMBASE, Chinese National Knowledge Infrastructure Database (CNKI), and the Wanfang Database. Other sources will also be searched like Google Scholar and gray literature. All databases mentioned above are searched from the start date to the latest version. Randomized controlled trials will be included which recruiting PCSD participants to assess the efficacy and safety of Chinese herbal medicines against controls (placebo or other therapeutic agents). Primary outcomes will include the size of PCSD, menstrual cycle, menstrual phase, menstrual volume, duration of disease, security index. Two authors will independently scan the searched articles, extract the data from attached articles, and import them into Endnote X8 and use Microsoft Excel 2013 to manage data and information. We will assess the risk of bias by Cochrane tool of risk of bias. Disagreements will be resolved by consensus or the participation of a third party. All analysis will be performed based on the Cochrane Handbook for Systematic Reviews of Interventions. The meta-analysis in this review will use RevMan 5.3 software.

**Results:**

: The study aims to evaluate the efficacy and safety of the treatment that Chinese herbal medicine for PCSD.

**Conclusion:**

: This study of the meta-analysis could provide evidence for clinicians and help patients to make a better choice.

**INPLASY registration number:**

INPLASY202090080

## 1 Introduction

Previous cesarean scar defect (PCSD), defined as the myometrium of uterus defects, is one of the late complications of cesarean delivery and the incidence is 19.4% to 88%.^[1–4]^ Because of the healing defect of uterine incision after cesarean operation, the scar would hinder menstruation and accumulate it in the depression, causing bleeding after intercourse, prolonging menstrual period, intermenstrual bleeding, dysmenorrhea, and other symptoms, and even lead to infertility. If a woman is pregnant again with the scar that does not cure, there is a greater probability of uterine rupture, bleeding in pregnancy because of the embryo implanting on caesarean scar, and other serious consequences. The pathological mechanism is still unclear because the scar is mainly located in the isthmus, some in the upper part of the cervix, affected by the original cesarean incision site and whether the cervix expansion or shortening and other factors.^[5]^ But the main causes may be the following:

1. The scar is at lower uterine segment where the cervical edge is thin and long, the uterine body edge is thick and short, so there is difference in thickness and contractility between the ends of the scar.^[6]^
2. The suture that can not be absorbed quickly causes ischemia, incision rupturing, and tissue necrosis.^[7]^
3. Because of endometriosis that occurs at the scar, the pressure led by endometrium fall off in menstrual period causes the scar break.^[8]^
4. Infection of uterine incision.
5. Affected by systemic factors or bad habits.

Cesarean delivery is one of the most common operations and its rate keeps increasing. In China, the rate is 50%^[9]^ which is much higher than the WHO's 15% limit, and as the promotion of the policy of a second child, the rate would be higher and the incidence of the disease would be higher, as a result, the incidence of the disease will continue to increase.

But there is no unified treatment for this disease and the corresponding treatment strategy is mainly selected according to the wishes and symptoms of the patients at present. The mainstream treatments are medication (like oral contraceptives or combination of estrogen and progesterone), intrauterine device, and surgery.^[10]^ But there are also some problems: oral contraceptives just can improve clinical symptoms in a short time and the recurrence rate is high after drug withdrawal.^[11]^ Surgery may be the best method to deal with the question, but the standard of surgical treatment has not been unified,^[12]^ and with the deepening of the understanding of PCSD, we find that different surgical treatment should be selected corresponding to different types of the disease and there would also be a greater risk of surgery complications like uterine perforation and damnification of bladder, etc. So further research is needed,^[13]^ more effective and comprehensive strategy is needed. Chinese herbal medicine is one of the most common treatments in traditional Chinese medicine (TCM), using the medicine extracted from botanical or mineral sources to cure diseases. In China and East Asia, Chinese herbal medicine has a history of thousands of years in the treatment of various diseases and it has been involved in the intervention of PCSD for decades, which greatly enriches the treatment of PCSD and achieves better curative effect like promoting recovery and relieving complications. The difference between human and experimental animals is that the human population is a complete mixture rather than test animals (same age, weight, sex, strain, etc). PCSD is also the result of several factors, including internal and external factors such as age, type of delivery, hypertension, shape of body, the ability of absorption of sutures, drinking, smoking status, etc. We must therefore take a holistic approach to different individuals and qualities. This is fully a close correspondence with the characteristics of TCM syndrome differentiation. As a significant method of TCM treatment, Chinese herbal medicine plays a increasingly important role in the treatment of PCSD, and in western countries, Chinese herbal medicine as a form of complementary medicine is increasingly accepted.^[14]^ Researches show that Chinese herbal medicine or the combination of Chinese herbal medicine and western medicine or surgery can resolve the clinical symptoms and quality of life better than the western medicine or surgery alone.^[15–26]^ However, based on the application of Western medicine or surgery, the efficacy and safety of TCM on PCSD still needs to be verified and no comprehensive assessments have been reported in recent years. Therefore, our aim is to collect the latest data of PCSD on Chinese herbal medicine, evaluate the therapeutic effect of Chinese herbal medicine, and support clinical decision-making.

## 2 Methods and analysis

### 2.1 Objectives and registration

This article will assess the efficacy and safety of Chinese herbal medicine on PCSD. The protocol has been registered in the International Platform of Registered Systematic Review and Meta-analysis Protocols (INPLASY) as INPLASY202090080. And the article will adhere to the Preferred Reporting Items for Systematic Reviews and Meta-Analyses Statement (PRISMA-P reporting guidelines).^[27]^

### 2.2 Eligibility criteria

#### 2.2.1 Types of studies

Randomized controlled trials (RCTs) in Chinese and English will be enrolled in this systematic review. Nonrandomized controlled trials (non-RCTs), quasirandomized controlled trials (qRCTs), cohort studies, reviews, experimental studies, expert experience, case reports, the data of the included study is missing or incomplete, and duplicate publications will be excluded.

#### 2.2.2 Types of participants

All participants with PCSD will be included regardless of their nationality, occupation, educational background, belief, age, body, or race.

#### 2.2.3 Patient and public involvement

This study has no patient and public involvement in consideration of this protocol for a systematic review

#### 2.2.4 Types of interventions

All kinds of Chinese herbal medicine will be included, there are no restrictions on the amounts of herbs, methods of administration, dosage, or duration of treatment. The comparisons will be either with other therapeutic agents or placebo.

### 2.3 Types of outcome measures

#### 2.3.1 Primary outcomes

1. The size of PCSD
*2.* Menstrual cycle
*3.* Menstrual phase
*4.* Menstrual volume
*5.* Duration of disease
*6.* Security index: general physical examination (temperature, pulse, respiration, blood, pressure), routine examination of blood, urine and stool, electrocardiogram, liver, and kidney function examination.

#### 2.3.2 Secondary outcomes

Symptoms (like lower abdominal pain) relieve.

### 2.4 Search methods

#### 2.4.1 Electronic searches

We will search the following databases for the identification of RCTs: PubMed, the Cochrane Library, Chinese Biomedical Literature Database (CB), Chinese Science and Technique Journals Database (VIP), Excerpt Medica Database (EMBASE), Chinese National Knowledge Infrastructure Database (CNKI), and the Wanfang Database. All the above databases will be searched from the available date of inception until the latest issue (November 2020).

#### 2.4.2 Other sources

We will search the reference lists of reviews and retrieve articles for additional studies on Google Scholar to identify further studies. We will include the literature published in journals and also “gray literature” such as degree theses and conference proceedings.

#### 2.4.3 Search strategy

Search strategy will follow the Cochrane handbook. The search strategy for PubMed is shown in Table 1, and similar strategies will be built and applied for other electronic databases.

**Table 1:** The search strategy for Pubmed.

Number	Search terms
#1	Previous cesarean scar defect[MeSH Terms]
	OR cesarean scar defect
#2	Traditional Chinese medicine[Title/Abstract]
	OR Chinese medicine [Title/Abstract]
	OR Chinese herbal medicine [Title/Abstract]
#3	RCT[Title/Abstract]
	OR randomized controlled trial[Title/Abstract]
#4	Efficacy[Title/Abstract] OR Safety[Title/Abstract]
#5	#1and #2 and #3 and #4

### 2.5 Data collection and analysis

#### 2.5.1 Selection of studies

**Figure 1:**
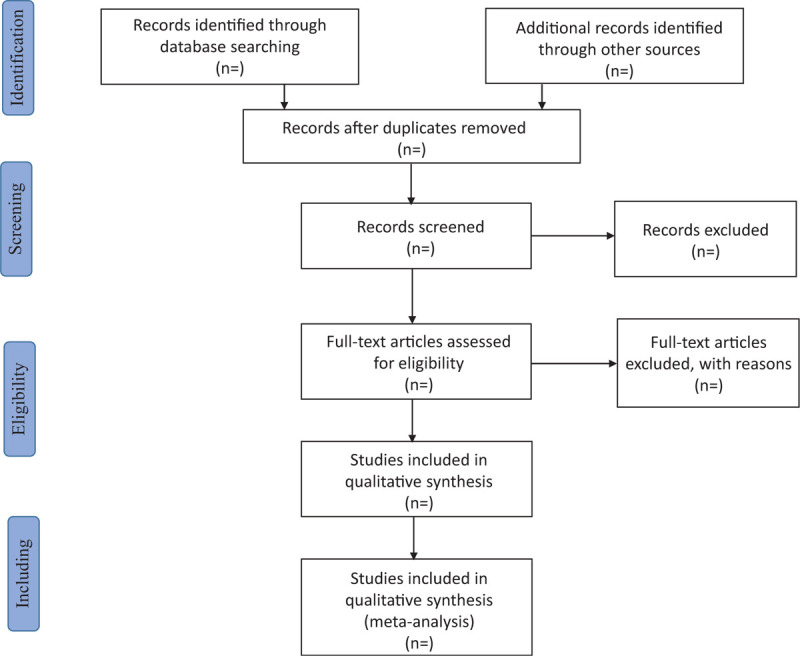
Flow chart of study selection.

Relevant literature will be obtained from the above databases, later imported into a database created by Endnote X8. Duplicate documents will be excluded through this process. And then the 2 review authors (JD and SL) will independently scan the titles abstracts and keywords of all articles identified from the electronic databases. Full-text articles will be scanned for all potentially relevant articles. If there is any disagreement on the selection of the article, it will be discussed with the third author (YP). The selection process will be shown in the Preferred Reporting Items for Systematic Review and Meta-analysis flow chart in Figure 1.

#### 2.5.2 Data extraction and management

For all studies included, 2 reviewers (CW and ZF) will extract relevant information independently. Microsoft Excel 2013 will be used for data and information management. The disagreement will be settled by another reviewer (MZ). We will extract for information as follows:

1. Basic information: titles, 1st author, corresponding author, publication time, country, and journal.
2. Participants’ characteristics: average age, location, sample size, inclusion and exclusion criteria.
3. Intervention method: treatment duration, study design, randomization, allocation concealment, and blinding methods
4. Outcomes: measures, primary and secondary outcomes, security indexes, and follow up.

#### 2.5.3 Assessment of the risk of bias in the included studies

Two authors (ZC and ZW) will use the Cochrane tool of risk of bias to assess the risk of bias independently. The disagreement will be settled by another reviewer (YJ). We will evaluate the following contents: selection bias (random sequence generation, and allocation concealment), performance bias (blinding of participants and personnel), detection bias (blinding of outcome assessment), attrition bias (incomplete outcome data), reporting bias (selective outcome reporting), and other bias (other sources of bias). Studies will be evaluated as high, low, and unknown.

#### 2.5.4 Measurement of the treatment effect

The meta-analysis in this review will use RevMan 5.3 software. Continuous variables will be reported as mean difference with 95% confidence intervals (CIs). For different measurement scales, we will use the standardized mean difference analysis with 95% CIs. Categorical variables will be summarized as risk ratios or odds ratios with 95% CIs. All analyses will be conducted in accordance with the Cochrane Handbook for Systematic Reviews of Interventions.^[28]^

#### 2.5.5 Units of analysis issues

All studies of parallel design will be included in this review. For cross-over trials, we will only analyze the data from first treatment period. For studies with multiple control groups, the unit of analysis will be used for each of the control groups.

#### 2.5.6 Dealing with missing data

If the information is missing or insufficient, we will contact the author of the study. If we fail to obtain sufficient data, we will exclude the study.

#### 2.5.7 Assessment of heterogeneity

Heterogeneity will be assessed by visual inspection of the forest and tested by standard Chi-squared statistic and a significance level of 0.1. Furthermore, the *I*^2^ statistic will be used to examine heterogeneity to quantify inconsistency. Fixed or random effects models will be performed in meta-analysis. If *I*^2^ > 0.5, the random effects models will be used.^[28]^

#### 2.5.8 Assessment of reporting biases

If 10 or more studies that we include in the meta-analysis, funnel plots and Egger test will be used to evaluate the reporting bias, like publication bias. We will assess the potential for small study bias by using funnel plots. The asymmetry of funnel plots will show the possible small research effects.^[29,30]^

#### 2.5.9 Subgroup analysis

If heterogeneity is detected, subgroup analysis will be performed to explore the differences in the methodologic quality, age, race/ethnicity, and types of Chinese medicine.

#### 2.5.10 Sensitivity analysis

Sensitivity analysis will be performed to examine the robustness of the result if there are sufficient studies included. The factors on effect are as follows:

- Methodologic quality: analysis will be performed excluding studies of poor methodologic quality.
- Sample size: analysis will be performed excluding small sample size studies.
- Diagnostic criteria: analysis will be performed in studies of the same diagnostic criteria.

#### 2.5.11 Confidence in cumulative evidence

The evidence level of the results will be assessed by a methodology based on the Grading of Recommendations Assessment, Development, and Evaluation. The evidence quality will be evaluated based on several factors including research limitations, effect consistency, imprecision, indirectness, and publication bias. The assessments will be categorized as high quality, medium quality, low quality, and very low quality.

## 3 Discussion

In recent years, the clinical RCTs of PCSD have been increasing; however, it is not enough satisfactory in the therapy of the disease. The clinicians have not been a consensus on the therapeutic principles and lack unified normalized standards. At present, the incidence of the disease is increasing, the impact on women is growing, but there are fewer reports than other gynecological diseases in related literature. Chinese medicine could help make up for the shortcoming of the treatments now. Chinese medicine, which a significant part of TCM possesses the characteristics of small side effects and simple and easy operation, has long been used to treat gynecological diseases. TCM has a profound rationale and rich clinical experience in the treatment of PCSD.^[31]^ The TCM treatment mainly achieves therapeutic effects by stimulating the body's qi and regulating the balance of qi and blood, yin, and yang.^[22]^ We have to admit the specific mechanism of TCM treatment of PCSD is not very clear, but clinical studies have demonstrated that TCM treatment of PCSD can relieve symptoms to some extent.^[32]^

Therefore, we will assess the efficacy and safety of TCM for the treatment of PCSD by using systematic review and meta-analysis. The results of this study can provide a possible ranking for TCM treatment of PCSD. We hope that these results will provide clinicians with the evidence for TCM treatment of PCSD and help clinicians to make the best choices for the treatment of patients.

### 3.1 Strengths and limitations of this study

Study selection, data extraction, and quality assessment will be performed independently by 2 researchers, which will ensure that all relevant studies are included without personal biases.

This study will focus on relevant clinical information used in clinical practice, which may facilitate the application of the review's findings to the clinical setting.

There may be high heterogeneity from the various evaluation standards in different prescription of Chinese herbal medicine.

Although this study will conduct a comprehensive search, it will not search for languages other than Chinese and English, which will lead to some bias.

### 3.2 Ethics and dissemination

Ethical approval is not required as this protocol is for a systematic review. In this study, participants are not recruited and data are not collected from participants. The review will be disseminated through peer-reviewed publications.

## Author contributions

**Conceptualization:** Jiashuai Deng, Yong Jiang.

**Data curation:** Jiashuai Deng, Sixuan Li, Changhong Wang.

**Formal analysis:** Jiashuai Deng, Zhipeng Fan, Mao Zhao.

**Methodology:** Jiashuai Deng, Yangzhi Peng, Zhaodi Wang.

**Project administration:** Jiashuai Deng, Zhaoxing Chen, Yuchang Jiang.

**Supervision:** Yong Jiang.

**Writing – original draft:** Jiashuai Deng, Sixuan Li.

**Writing – review & editing:** Jiashuai Deng, Yangzhi Peng, Yong Jiang.

## References

[1] Uppal T Lanzarone V Mongelli M Sonographically detected caesarean section scar defects and menstrual irregularity. J Obstet Gynaecol 2011;31:413–6. 21627425 10.3109/01443615.2011.577252

[2] Borges LM Scapinelli A de Baptista Depes D Findings in patients with postmenstrual spotting with prior cesarean section. J Minim Invas Gyn 2010;17:361–4. 10.1016/j.jmig.2010.02.007 20417429

[3] Osser VO Valentin L Risk factors for incomplete healing of the uterine incision after caesarean section. BJOG 2010;117:1119–26. 20604776 10.1111/j.1471-0528.2010.02631.x

[4] Ofili-Yebovi D Ben-Nagi J Sawyer E Deficient lower-segment cesarean section scars: prevalence and risk factors. Ultrasound Obstet Gynecol 2008;31:72–7. 18061960 10.1002/uog.5200

[5] Cho CE Norman M Cesarean section and development of the immune system in the offspring. Am J Obstet Gynecol 2013;208:249–54. 22939691 10.1016/j.ajog.2012.08.009

[6] Levi E Cantillo E Ades V Immediate postplacental IUD insertion at cesarean delivery: a prospective cohort study. Contraception 2012;86:102–5. 22264666 10.1016/j.contraception.2011.11.019

[7] Wu YT Xia HW Zeng XR Clinical analysis of operation on vagina of cesarean section scar diverticulum. Nat Med Front China 2011;06:51–2.

[8] He M Chen M Xie H Transvaginal removal of ectopic pregnancy tissue and repair of uterine defect for caesarean scar pregnancy. BJOG 2011;118:1136–9. 21481146 10.1111/j.1471-0528.2011.02891.x

[9] Hellerstein S Feldman S Duan T China's 50% caesarean delivery rate: is it too high? BJOG 2015;122:160–4. 25138909 10.1111/1471-0528.12971

[10] Shi Q Wang QY Clinical status and progress of PCSD. Matern Child Health Care China 2014;29:3530–2.

[11] Li LP Liu J Lu XH Comparative study on the effect of laparoscopic scar resection and repair and estrogen and progesterone in 78 cases of PCSD. Matern Child Health Care China 2015;30:4407–9.

[12] Marotta ML Donnez J Squifflet J Laparoscopic repair of post-cesarean section uterine scar defects diagnosed in nonpregnant women. J Minim Invasive Gynecol 2013;20:386–91. 23357466 10.1016/j.jmig.2012.12.006

[13] Li J Bai WP The treatment of previous cesarean scar defect. J Int Obstet Gynecol 2017;44:543–6.

[14] Hu J Zhang J Zhao W Cochrane systematic reviews of Chinese herbal medicines: an overview. PLoS One 2011;6:e28696. 22174870 10.1371/journal.pone.0028696 PMC3235143

[15] Lv X Wang XH Chen L Observation of the clinical effect of traditional Chinese medicine cycle therapy on prolonged menstrual period caused by uterine incision diverticulum. J Pract Tradit Chin Med 2020;36:830–2.

[16] Yuan Y The clinical observation of Huayu Xiaozheng decoction treating previous cesarean scar defect for patients of blood statis: Fujian University of Traditional Chinese Medicine; 2019.

[17] Yang L Clinical study of Yiqi Huayu Shengji Decoction in the treatment of cesarean scar diverticulum with Qi Deficiency and Blood Stasis type: Fujian University of Traditional Chinese Medicine; 2019.

[18] Ding N Clinical observation of Qi-Tonifying and Heat-clearing and Blood-stasis-removing method treating bradymenorrhea with PCSD: Nanjing University of Chinese Medicine; 2019.

[19] Wang B Analysis of the curative effect of Baogong Zhixue Granule in the treatment of prolonged menstruation of uterine incision diverticulum. Medical Diet and Health 2019;18:36–7.

[20] Wang SY Observation of the clinical efficacy of ShenGuiZhiLou decoction in the treatment of Previous Cesarean Scar Defect (PCSD): Hunan University of Chinese Medicine; 2018.

[21] Jing SJ Clinical observation of blood stasis of menstrual extension caused by the treatment of the self-dispensing blood stasis to the soup in the treatment of PCSD: Changchun University of Chinese Medicine; 2018.

[22] Li ZH Comparative analysis of clinical efficacy of Shenqi Sihua Decoction and Marvelon in the treatment of prolonged menstruation caused by PCSD. J Sichuan Tradit Chin Med 2017;35:120–2.

[23] Zhong YH Clinical observation of the two-steps traditional Chinese medicine in treating previous cesarean scar defect: Zhejiang Chinese Medical University; 2016.

[24] Zhong YH Clinical observation of the two-steps traditional Chinese medicine in the treatment of prolonged menstruation caused by PCSD. Zhejiang J Tradit Chin Med 2016;51:833–4.

[25] Wang QH Xu MC Clinical analysis of oral contraceptives combined with traditional Chinese medicine in treatment of 56 cases of uterine incision diverticulum after cesarean section. Med Innov China 2015;12:92–4.

[26] Qi Q Clinical obersvation of Shen-Qi Four Flower Decoction treating uterus incision lacuna with bradymenorrhea: Hunan University of Chinese Medicine; 2014.

[27] Moher D Shamseer L Clarke M Preferred reporting items for systematic review and meta-analysis protocols (PRISMA-P) 2015 statement. Syst Rev 2015;4:1. 25554246 10.1186/2046-4053-4-1 PMC4320440

[28] Cumpston M Li T Page MJ Updated guidance for trusted systematic reviews: a new edition of the Cochrane Handbook for Systematic Reviews of Interventions. Cochrane Database Syst Rev 2019;10:ED000142. 31643080 10.1002/14651858.ED000142 PMC10284251

[29] Lau J Ioannidis JP Terrin N The case of the misleading funnel plot. BMJ 2006;333:597–600. 16974018 10.1136/bmj.333.7568.597 PMC1570006

[30] Sterne JA Egger M Smith GD Systematic reviews in health care: Investigating and dealing with publication and other biases in meta-analysis. BMJ 2001;323:101–5. 11451790 10.1136/bmj.323.7304.101 PMC1120714

[31] Zhong YH Zhang Q The clinical experience of professor Zhang Qin in treating menostaxis caused by previous cesarean scar defect. J Zhejiang Chin Med Univ 2016;40:203–5.

[32] Liang XL Hong LM Progress in the Treatment of previous cesarean scar defect with traditional Chinese and Western medicine. Chin Foreign Med Res 2020;18:183–5.

